# Diversity in scarcity: financial scarcity uniquely impairs success-seeking beyond a universal surge in failure-avoidance

**DOI:** 10.3389/fpsyg.2026.1870851

**Published:** 2026-09-28

**Authors:** Guiping Dou, Ying Li, Min Zhang, Dan Pan, Fang Liu

**Affiliations:** 1 Department of Education Science, Zhoukou Normal University, Zhoukou, China; 2 Faculty of Psychology, South China Normal University, Guangzhou, China

**Keywords:** consistency and diversity, financial scarcity, time scarcity, motivation to avoid failure, motivation to pursue success

## Abstract

**Purpose:**

Social life is fundamentally shaped by how individuals perceive and respond to resource constraints. Although the “scarcity mindset theory” suggests a general cognitive cost of deprivation, research on how different contextual factors, especially time and financial scarcity, shape motivation structure remains limited. This study investigates the divergent effects of these two forms of scarcity on achievement motivation, specifically distinguishing between the motivation to pursue success (Ms) and the motivation to avoid failure (Mf).

**Methods:**

We employed a multi-study approach across three studies. Study 1 (*N* = 200) was a questionnaire survey designed to establish the initial ecological correlation between naturally occurring scarcity perceptions and motivational orientations. Study 2 (*N* = 180) used an unconstrained recall manipulation to probe causality; its results revealed that relatively high, unbuffered ecological baselines can complicate the specific effects of scarcity. To isolate the core psychological construct, Study 3 (*N* = 160) employed an orthogonal manipulation that anchored non-target resources at a neutral, zero-scarcity baseline, thereby disentangling general ecological strain from the unique psychological experience of each specific resource scarcity.

**Results:**

Our findings across the three studies support a “Convergent-Divergent” model of social cognition under scarcity. (1) Convergence: Both time scarcity and financial scarcity consistently enhance individuals’ motivation to avoid failure. (2) Divergence: The two forms of scarcity had different effects on the motivation to pursue success. Financial scarcity significantly suppressed the motivation to pursue success across both natural and controlled experimental contexts, whereas time scarcity did so only when experienced within an unbuffered, demanding natural context (Study 2; a pattern reflected by the control group’s naturally high baseline of concurrent scarcity pressures); when isolated at a neutral baseline (Study 3), time scarcity showed no significant suppressive effect, leaving the drive for achievement intact.

**Conclusion:**

The findings of this study reveal asymmetric effects of the motivational system under different resource-scarcity conditions and indicate that the psychological impact of scarcity is highly context-dependent. These findings have implications for motivational interventions and organizational management practices targeting different groups facing adversity.

## 1 Introduction

Individuals are not passive observers of the social environment; they are active agents who continuously interpret and respond to the external world through a cognitive lens shaped by their resource contexts (Bandura, 1986). Within social cognitive psychology, developing an understanding of the “cognitive architecture” that underpins such adaptive social functioning is central to explaining the mechanisms that generate human behavior (Kahneman, 2011). Among various environmental variables, scarcity of resources is a fundamental contextual factor that shapes human cognition and behavior (Mani et al., 2013; Mullainathan and Shafir, 2013).

In the fast-paced and highly competitive environment of modern society, the experience of scarcity has become pervasive in daily life (de Bruijn and Antonides, 2022). Whether facing the financial pressure of living paycheck to paycheck or struggling to meet a series of looming deadlines, individuals increasingly perceive a gap between what they possess and what they need, desire, or aspire to achieve (Zhao and Tomm, 2018). This disparity between “having” and “needing” constitutes the experiential core of the scarcity mindset (Cannon et al., 2019), which is a psychological state whose cognitive consequences have been extensively theorized.

Social cognitive theory posits that human behavior emerges from continuous, reciprocal interactions between personal factors (e.g., beliefs, goals) and environmental situations (Bandura, 1999). Viewed from this perspective, resource scarcity is by no means reducible to an objective lack of resources; rather, it constitutes a psychologically constructed state that reconfigures cognition and behavior (Mullainathan and Shafir, 2013). The overarching framework for explaining this process is scarcity theory. The theory proposes that any form of scarcity, be it of financial, time, or even calories, captures attention and creates a “cognitive tunnel.” This tunneling forces the mind to concentrate substantial resources on the immediate unmet need, a response that can enhance efficiency in managing the pressing problem in the short run (Shah et al., 2012) but incurs considerable long-term costs, including depleted cognitive bandwidth, diminished executive control, and impaired long-term planning (Mani et al., 2013). Through the lens of social cognition, scarcity effectively operates as a contextual prime that heightens the accessibility of certain goals and strategies while inhibiting others, thereby generating a “hot” cognitive state in which short-term problem-solving takes precedence over extended strategic thinking (Bargh and Chartrand, 1999; Higgins, 1996; Mullainathan and Shafir, 2013).

Yet, although scarcity theory furnishes a general model for understanding the cognitive consequences of resource shortfalls, the “generality hypothesis” embedded deep in its theoretical core warrants closer scrutiny (de Bruijn and Antonides, 2022; Mullainathan and Shafir, 2013). This hypothesis tends to treat scarcity as a monolithic construct, presuming that the depletion of qualitatively different resources—such as time and financial—yields functionally equivalent cognitive effects (Cannon et al., 2019; Mullainathan and Shafir, 2013; Shah et al., 2012). Such an assumption risks obscuring differences in how distinct resource types shape psychological functioning by placing a lopsided emphasis on their shared mechanisms (Cannon et al., 2019; Dhar and Wertenbroch, 2000). Human behavior, after all, is not simply the product of internal traits or a generic response pattern to cognitive load; rather, it emerges from the dynamic interplay between specific contextual features and the cognitive systems that individuals deploy to interpret them (Mischel and Shoda, 1995). Social cognitive processes are sensitive to the self-evaluative implications embedded within a given situation (Festinger, 1954). Therefore, to fully capture the psychological imprint of scarcity, one must extend the analytic focus to the unique social and psychological meanings carried by the scarce resource itself (Kruglanski et al., 2013; Trope and Liberman, 2010).

The foregoing discussion leads to a key theoretical question: Although different types of resource scarcity share the basic feature of inducing cognitive load, do they differentially reconfigure the architecture of human motivation? The present research addresses this question by focusing on achievement motivation, a cornerstone of human striving. Achievement motivation theory supplies an analytical framework for this purpose. Rooted in the pioneering work of Atkinson (1957) and McClelland (1961), the theory’s central postulate is that an individual’s tendency to engage in achievement-oriented activity is a joint function of two competing and independent motivational systems: the motivation to pursue success (Ms) and the motivation to avoid failure (Mf).

Specifically, Ms. constitutes an approach-oriented system. It is characterized by heightened attentiveness to positive potential outcomes such as mastery, growth, competence, and attainment. Individuals high in Ms. gravitate toward tasks of intermediate success probability, as these afford the greatest sense of accomplishment. They tend to be optimistic, to engage in calculated challenge-taking, and to exhibit greater persistence when pursuing rewards in the face of difficulty (Atkinson, 1957). At the level of social functioning, Ms. is closely linked with proactivity, leadership emergence, innovative behavior, and a keen attunement to opportunities for advancement (Elliot and McGregor, 2001; Frese and Fay, 2001). The core resource that sustains this system is perceived self-efficacy and the perceived attainability of a desirable future state (Bandura, 1997; Carver and Scheier, 1998).

In contrast, Mf is an avoidance-oriented system. It is characterized by preemptive vigilance toward negative potential outcomes, such as the exposure of incompetence, the experience of embarrassment, and the risk of social rejection. Individuals high in Mf devote their psychological resources primarily to guarding against failure. In selecting tasks, they gravitate toward either extremely easy ones (where success is guaranteed) or very difficult ones (where failure cannot be diagnosed as indicative of their ability) (Atkinson, 1957). This system typically triggers anxiety and a state of heightened alertness, steering individuals toward conservative, low-risk strategies (Elliot and McGregor, 2001). In interpersonal contexts, Mf is tightly bound to social conformity, performance anxiety, and a defensive posture organized around loss prevention (Gable et al., 2000). Its activation is contingent on the perception of potential threat and vulnerability to the self.

Ms. and Mf are not polar ends of a single continuum; they are qualitatively distinct regulatory systems that can be activated independently or co-activated (Atkinson, 1957; Elliot and McGregor, 2001). An individual’s ultimate behavioral orientation in any given achievement situation depends on the relative strength of these two motives. A person may present as a confident achiever (high Ms., low Mf), an ambitious yet anxious over-striver (high Ms., high Mf), or any number of other configurations (Atkinson, 1957). This dual-system architecture furnishes a theoretical testing ground for examining the psychological effects of scarcity (Kruglanski et al., 2013): Does scarcity exert a uniform influence on both motivational systems by generally increasing cognitive load? Or does it, instead, operate with greater nuance, differentially regulating each system according to the specific properties of the scarce resource—most importantly, the social meanings and evaluative standards embedded within it?

Addressing this question demands a closer analysis of the distinct social-cognitive meanings attached to different resource types (Kruglanski et al., 2013; Trope and Liberman, 2010). The social meaning and psychological representation of a resource may be as consequential as its objective absence. Although both time scarcity and financial scarcity constitute stressors that impose cognitive load, they convey different signals along the dimensions of social and self-evaluation (Cannon et al., 2019; Mullainathan and Shafir, 2013). In the modern social context, wealth functions as the most potent and broadly commensurable metric of social status and personal worth (Liang et al., 2025). An acutely quantifiable social signal, it readily invites direct and often painful social comparisons (Boyce et al., 2010; Buttrick and Oishi, 2023) and serves as a symbol of social standing, personal competence, and the “ticket” to achievement (Pfund et al., 2024). For this very reason, a lack of financial resources is seldom experienced as a neutral state of deficiency; rather, it is frequently internalized as a clear signal of personal inadequacy, low social rank, and blocked goal pathways (Park et al., 2017). This internalization process can provoke threatening social comparisons (Thulien et al., 2018) that, in turn, erode perceived social standing and self-efficacy (Sheehy-Skeffington, 2020).

Self-efficacy remains a central proximal determinant of Ms. in recent empirical research (Basileo et al., 2024; Hernandez-Perez and Cruz Rambaud, 2025). The drive to innovate, to accept challenge, and to pursue ambitious goals is predicated on a positive belief in one’s own prospect of succeeding (Li et al., 2024). When the signal broadcast by financial scarcity is interpreted as an indication that one is “falling behind” in competitive terms or that routes to success are blocked, this belief system may come under threat (Chakraborty et al., 2025). The ensuing feelings of shame, powerlessness, and diminished prospects are theorized to dampen the psychological impetus for approach-oriented behavior, potentially yielding a systematic attenuation of Ms. (Liu et al., 2024; Vohs et al., 2006). Following this logic, we reason that financial scarcity not only activates the defensive Mf system in response to the potential threat of economic hardship, but also may exert a distinct suppressive effect on the appetitive Ms. system, potentially by eroding the perceived feasibility and desirability of success.

In contrast, time scarcity has been socially constructed with different meanings in contemporary society. Across numerous professional domains and social circles, “extreme busyness” has evolved into a conspicuous badge of high status. Time poverty is frequently decoded as an overt signal that an individual is in high demand, highly productive, and situated at a critical juncture within an organization. A person who complains of a “packed schedule, without a free moment” is often simultaneously signaling that they are in high demand and occupy a valuable position in the labor market and the social hierarchy (Bellezza et al., 2017; Cannon et al., 2024). This distinctive social construction of time scarcity holds theoretical implications for its effects on achievement motivation (Monga et al., 2022). To be sure, looming deadlines and an overloaded schedule are undeniable stressors that are likely to activate the defensive Mf system by raising cognitive load (Shah et al., 2012). Yet they do not necessarily carry the same freight of negative self-evaluation as financial deprivation (So, 2018; Wu et al., 2025). Subjectively experiencing a shortage of time does not intrinsically trigger a sense of personal failure, a perception of low social standing, or a corrosion of self-efficacy (So, 2018). On the contrary, a moderate level of time pressure may even reinforce one’s sense of competence and social importance. Hence, even though time pressure heightens the need for vigilance and thereby Mf, there is little theoretical basis for expecting it to suppress Ms. The ambition to succeed may remain intact even when immediate behavioral strategies become more cautious. This inference stands in theoretical counterpoint to the “dual-system imbalance” precipitated by financial scarcity.

Synthesizing the preceding analysis, the current research aims to investigate whether the cognitive mechanisms that underpin Ms. and Mf operate in a differentiated manner under the distinct contextual pressures of time scarcity and financial scarcity. By moving beyond the generality hypothesis embedded in scarcity theory, we seek to construct a more refined theoretical model that specifies how particular environmental contexts exert differential effects on the formation of social attitudes and goal-directed behavior. To this end, we propose a Convergent-Divergent model, the core hypotheses of which are as follows:

*Hypothesis 1 (Convergence)*: Both time scarcity and financial scarcity will significantly increase participants’ Mf relative to a control condition.

*Hypothesis 2 (Divergence)*: Financial scarcity will significantly decrease participants’ Ms. relative to a control condition.

*Hypothesis 3 (Divergence)*: Time scarcity will not significantly decrease participants’ Ms. relative to a control condition.

To test this theoretical model, we designed and executed a systematic investigation comprising three sub-studies. Study 1 used a questionnaire survey to examine the ecological associations between self-reported scarcity and achievement motivation, thereby establishing a correlational foundation at the phenomenological level. Studies 2 and 3 adopted an experimental approach to manipulate perceived scarcity in order to probe causality. Through this progressively layered research design, the present investigation strives to bridge the explanatory gap between the external social-structural environment and internal motivation, thereby enriching our understanding of the factors that shape social life and adaptive human functioning.

Across all three studies, we employed path analysis rather than separate multiple regressions or ANOVA-based contrasts for two reasons. First, path analysis estimates effects on both dependent variables (Ms and Mf) simultaneously, while modeling their inherent negative covariance. This yields more accurate standard errors compared to separate multiple regressions. Second, a consistent analytic framework across the correlational study (Study 1), the three-group experiment (Study 2), and the four-group experiment (Study 3) enables direct cross-study comparison of effect sizes and coefficient patterns, which is essential for evaluating the convergent-divergent model.

All models were estimated in Mplus 8.0 (Muthen and Muthen, 2017) using maximum likelihood estimation. For the experimental studies, dummy-coded variables representing the conditions were entered simultaneously as predictors, with the control group coded 0 on all dummies. Full coding details are provided in each study’s Method section. Because all path models are just-identified (saturated, df = 0), fit indices are mathematically determined and are reported for transparency rather than as evidence of model fit.

Table 1 in Supplementary Material C summarizes the demographic characteristics of the three samples. The samples were broadly comparable in terms of age (means ranging from 30.61 to 33.69 years) and gender distribution (56–61% female across studies). Educational attainment was highly consistent, with mean levels corresponding approximately to a bachelor’s degree (M = 5.01–5.23 on a 7-point scale). Household income fell within the middle-income brackets across all three studies (M = 6.77–7.62 on a 10-point scale), though Study 3 showed a slightly lower mean income compared to Studies 1 and 2. Subjective socioeconomic status (SSS), measured via the MacArthur Scale, was near the midpoint in all three studies (M = 5.27–5.70), with adequate variability (SD = 1.21–1.24). Overall, the demographic profiles support the cross-study comparability of the findings. Note that SSS was included as a covariate only in Study 1, as Studies 2 and 3 employed experimental designs in which participants were randomly assigned to scarcity conditions. In these experimental studies, SSS was measured for sample description but was not included in the path models, consistent with the logic that randomization obviates the need for covariate control of baseline differences in social rank.

**Table 1:** Results of variable descriptive statistics and correlation analysis in study 1.

Variable	1	2	3	4	5	6	7
1. TS	1						
2. FS	0.267^***^	1					
3. Ms	−0.139^†^	−0.465^***^	1				
4. Mf	0.311^***^	0.662^***^	0.730^***^	1			
5. Income	−0.041	−0.404^**^	0.281^**^	−0.241^**^	1		
6. Edu	−0.126	−0.310^**^	0.364^**^	−0.320^**^	0.237^**^	1	
7. SSS	−0.213^**^	−0.395^**^	0.394^**^	−0.332^**^	0.379^**^	0.166^**^	1
M	4.587	3.404	3.915	2.262	7.620	5.230	5.710
SD	1.831	1.793	0.864	1.060	1.369	0.683	1.206

TS, Time scarcity; FS, Financial scarcity; Ms, Achievement motivation to pursue success; Mf, Achievement motivation to avoid failure. †indicates *p* = 0.05, ^*^indicates *p* < 0.05, ^**^indicates *p* < 0.01, ^***^indicates *p* < 0.001, the same below.

## 2 Study 1: the relationship between the sense of scarcity of time and financial and achievement motivation

### 2.1 Method

#### 2.1.1 Participants

Data were collected from 200 participants recruited through “Credamo” (a Chinese platform comparable to Mturk or Prolific). The sample had a mean age of 32.68 years (SD = 6.67, range = 19–56) and consisted of 78 men and 122 women.

#### 2.1.2 Measurement

##### 2.1.2.1 Sense of time/financial scarcity

Participants rated their perceptions of time and financial scarcity using the Resource Scarcity Scale (Roux et al., 2015). Time scarcity was measured with four items (e.g., “I don’t have enough time”), and financial scarcity was measured with four parallel items (e.g., “I don’t have enough money”). Responses were collected on a 7-point Likert scale (1 = strongly disagree to 7 = strongly agree), with a six-month recall period. Cronbach’s α coefficients were 0.949 and 0.945 for the time and financial scarcity, respectively. The complete instrument in the original Chinese and English translation is provided in the Supplementary Materials A.

##### 2.1.2.2 Achievement motivation

We used the short version of the Achievement Motivation Scale revised by Tang and Lu (2013), which was adapted from the original scale developed by Norwegian psychologists Nygård and Gjesme (1973). The scale comprises 12 items—six measuring Ms. (e.g., “I am drawn to challenging tasks”) and six measuring Mf (e.g., “When the outcome is uncertain, I worry about failure”). Responses were recorded on a 5-point Likert scale ranging from 1 (strongly disagree) to 5 (strongly agree). The Cronbach’s α were 0.912 for the success pursuit dimension and 0.937 for the failure avoidance dimension. The complete instrument in the original Chinese and English translation is provided in the Supplementary Materials B.

##### 2.1.2.3 Control variables

To examine whether perceived financial scarcity predicts motivational orientations independently of subjective socioeconomic status (SSS) and the other socioeconomic indicators, we controlled for educational level, income, and SSS. Perceived resource scarcity and SSS, although both grounded in one’s objective socioeconomic conditions, capture conceptually distinct psychological processes. Perceived scarcity reflects a self-referent, vertical comparison between what one has and what one needs or desires. SSS reflects a social-referent, horizontal comparison of one’s standing relative to others (Adler et al., 2000). SSS was measured using the MacArthur Scale of Subjective Social Status (Adler et al., 2000), which asks participants to indicate their relative social status on a 10-rung ladder (1 = lowest, 10 = highest). Controlling for SSS thus addresses a specific theoretical question: it allows us to examine whether perceived financial scarcity predicts motivational orientations independently of the generalized psychological burden of macro-level social rank. Income and education were included as objective SES covariates that may affect achievement motivation. Educational level was categorized into 7 groups ranging from “primary school or below” to “postgraduate and above”. Income was divided into ten ranges (1 = “less than CNY 3,600”; 10 = “CNY 500,001 and above”).

### 2.2 Data analysis

We first conducted descriptive statistics using SPSS 26.0, followed by path analysis performed with Mplus 8.0.

### 2.3 Results

#### 2.3.1 Descriptive statistics

Table 1 displays the descriptive statistics and zero-order correlations among the variables. The correlation results showed a positive relationship between time scarcity and Mf (*r* = 0.311, *p* < 0.001), whereas the negative correlation with Ms. fell just at the threshold of significance (*r* = −0.139, *p* = 0.05). Diverging from this pattern, financial scarcity was negatively correlated with Ms. (*r* = −0.465, *p* < 0.001) and positively correlated with Mf (*r* = 0.662, *p* < 0.001). Furthermore, income, educational level, and SSS all show a significant positive correlation with Ms. (*r*s = 0.281, 0.364, and 0.394, *p*s < 0.01), and a significant negative correlation with Mf (*r*s = −0.241, −0.320, and −0.332, *p*s < 0.01). More descriptive statistics for other variables are presented in Table 1 of Supplementary Material C.

#### 2.3.2 Path analysis

We tested our theoretical model using path analysis (Wright, 1960), which allowed for the simultaneous estimation of effects on the two dependent variables: Ms. and Mf. The covariate-adjusted path model included all observed covariates as simultaneous predictors of Ms. and Mf, with their residual covariance freely estimated. Consistent with the framework described in the Introduction, we treat this adjusted model and the unadjusted model reported in Supplementary Material C (Table 2) as complementary analyses addressing different theoretical questions. The model was just-identified (saturated, df = 0), and therefore fit indices are mathematically determined (*χ^2^* = 0, CFI = 1.000, RMSEA = 0.000, SRMR = 0.000). The model accounted for 32.7% of the variance in Ms. (*f^2^* = 0.49) and 48.0% of the variance in Mf (*f^2^* = 0.92). Complete path coefficients, including 95% confidence intervals, are presented in Table 2. Of the covariates, educational level significantly predicted both Ms. and Mf, SSS significantly predicted Ms., and income was not significant. The path coefficients (Figure 1) showed that both time and financial scarcity positively predicted Mf. A key divergence emerged for Ms.: time scarcity was not a significant predictor, whereas financial scarcity negatively predicted it.

**Table 2:** Path coefficients for the study 1 path model.

Path	B	*β*	*SE*	*z*	*p*	95% CI for B
Scarcity → Ms						
TS → Ms	0.007	0.016	0.030	0.243	0.808	[−0.052, 0.066]
FS → Ms	−0.146	−0.302	0.034	−4.315	<0.001	[−0.212, −0.079]
Scarcity → Mf						
TS → Mf	0.066	0.113	0.032	2.021	0.043	[0.002, 0.129]
FS → Mf	0.350	0.592	0.036	9.615	<0.001	[0.278, 0.421]
Covariates → Ms						
Income → Ms	0.006	0.009	0.042	0.132	0.895	[−0.078, 0.089]
Edu → Ms	0.304	0.240	0.082	3.695	<0.001	[0.143, 0.465]
SSS → Ms	0.167	0.233	0.049	3.442	<0.001	[0.072, 0.262]
Covariates → Mf						
Income → Mf	0.041	0.053	0.046	0.904	0.366	[−0.048, 0.131]
Edu → Mf	−0.184	−0.118	0.089	−2.073	0.038	[−0.357, −0.010]
SSS → Mf	−0.070	−0.079	0.052	−1.333	0.182	[−0.172, 0.033]
Residual covariance						
Ms ↔ Mf	−0.349	−0.647	0.045	−7.679	<0.001	[−0.437, −0.260]

**Figure 1:**
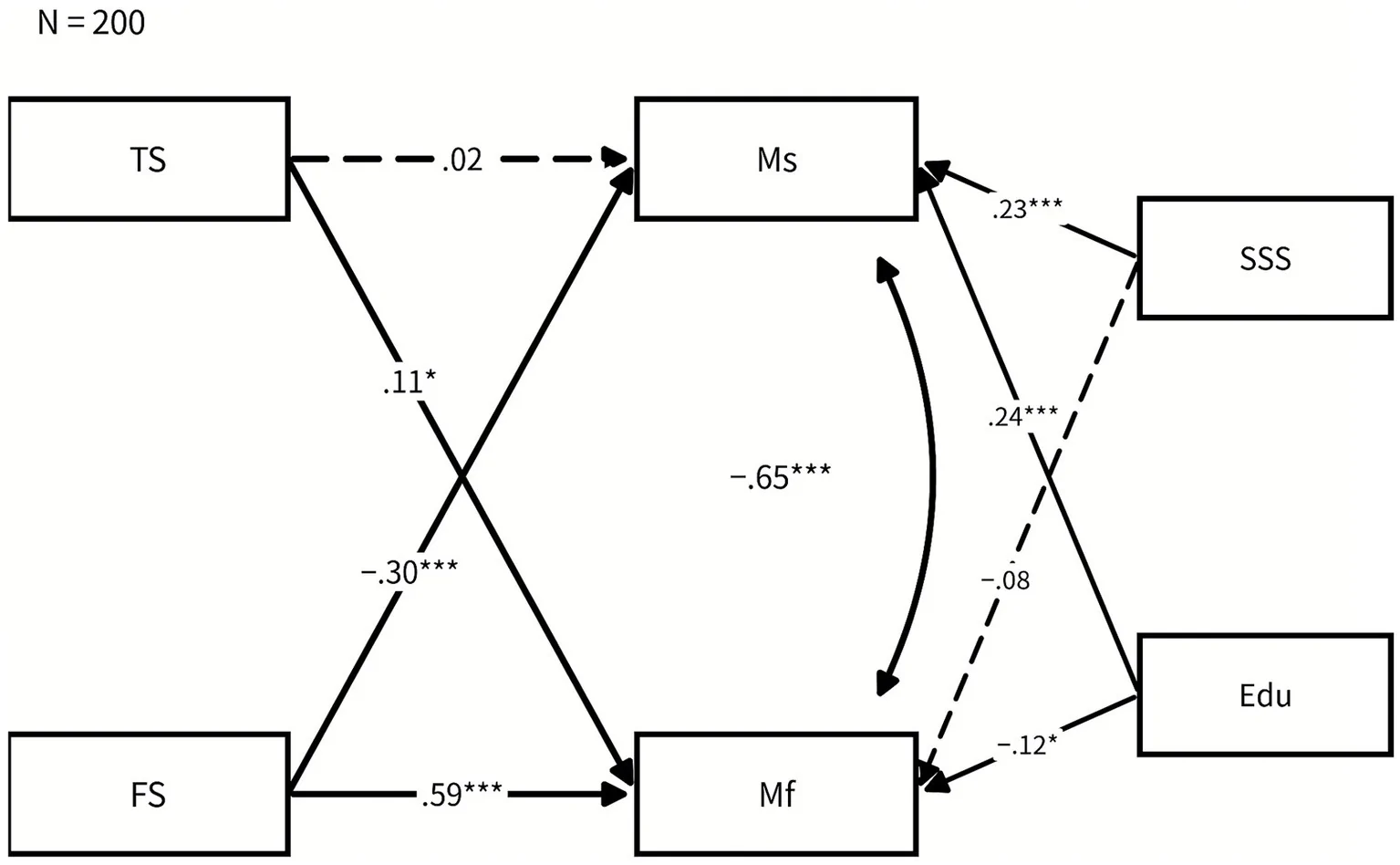
Results of path analysis in study 1 (*N* = 200). Rectangles represent measured variables; single-headed arrows represent hypothesized directional paths from scarcity perceptions to motivational orientations, with standardized path coefficients (STDYX) shown along each arrow. Solid arrows indicate statistically significant paths; the dashed arrow indicates a non-significant path. The curved double-headed arrow represents the residual covariance between Ms. and Mf. *p < 0.05. **p < 0.01. ***p < 0.001.

It is important to clarify the theoretical meaning of these estimates. The SSS-adjusted model should not be regarded as the only appropriate test; rather, it addresses a specific question, namely whether financial scarcity predicts Ms. independently of SSS. Because financial scarcity and subjective social standing share meaningful conceptual variance in real-world settings, controlling for SSS may remove theoretically meaningful variance if financial scarcity affects motivation partly through lowering perceived social standing (Schisterman et al., 2009). To examine this possibility transparently and to assess the robustness of our findings, we also estimated an unadjusted model excluding covariates (see Table 2 of Supplementary Material C). This dual-model approach clarifies the intertwined nature of scarcity and social rank versus the distinct effect of specific resource deprivation. These two models are therefore complementary rather than competing, and neither is treated as the principal analysis.

As shown in Table 2 of Supplementary Material C, the differences between the estimates are theoretically informative. In the unadjusted model, the effect of financial scarcity on Ms. was stronger (*β* = −0.460). This unadjusted estimate captures the broader ecological reality where financial scarcity and perceived low social standing jointly suppress motivation. When covariates were introduced, this effect showed partial attenuation (*β* = −0.302) but remained statistically significant (*p* < 0.001). This attenuation indicates that SSS shares theoretically meaningful variance with financial scarcity. However, this stable residual effect is theoretically relevant to our comparative framework: it indicates that financial scarcity suppresses Ms. independently of SSS and objective SES, highlighting the distinct psychological effect of specific resource deprivation.

In contrast, the effect coefficient of time scarcity on Ms. remained non-significant in both models. Both types of scarcity significantly and positively predicted Mf (*β* changed from 0.145 to 0.113 for time scarcity, and *β* changed from 0.623 to 0.592 for financial scarcity), with minimal attenuation.

### 2.4 Brief discussion

The results of Study 1 were consistent with our convergent-divergent model. Both forms of scarcity were associated with higher Mf, but only financial scarcity was linked to lower Ms. Of note, this suppressive effect of financial scarcity on Ms. remained significant even when controlling for subjective social standing, indicating an independent psychological effect of specific resource deprivation. While these findings are ecologically valid, their correlational nature precludes causal inference. It is possible, for example, that a low drive for success leads to poorer financial outcomes, rather than the reverse. To establish causality, an experimental approach was necessary.

## 3 Study 2: the effects of perceived time scarcity and financial scarcity on achievement motivation

To examine the causal relationships suggested by the correlational findings of Study 1, Study 2 adopted a one-way between-subjects design (time scarcity group, financial scarcity group, and control group). Participants were randomly assigned to one of the conditions. The procedure involved an experimental manipulation, followed by a manipulation check, and finally, the measurement of achievement motivation.

### 3.1 Method

#### 3.1.1 Participants

*A priori* power analysis was conducted using G*Power (Faul et al., 2007). It indicated that a sample size of 158 would be required to achieve 80% power for detecting a medium effect size (*f* = 0.25) at an alpha level of 0.05 (two-tailed). Accordingly, we recruited 180 participants via “Credamo,” with 60 individuals assigned to each group. The sample had a mean age of 33.69 years (SD = 6.89, range = 22–63) and consisted of 70 men and 110 women.

#### 3.1.2 Manipulation

We employed the contextual recall paradigm (Roux et al., 2015) to induce sense of scarcity. Participants in the experimental groups were asked to list two instances in which they experienced a shortage of either time or financial resources and to describe their experiences, reasons, and feelings in those situations. Participants in the control group were instructed to recall and describe in detail two ordinary events from the past week.

Following the recall task, participants completed manipulation check items designed to assess their current, state-level perceived scarcity. The instructions explicitly asked participants to indicate how they felt “right now.” All participants, regardless of experimental condition, responded to both time-scarcity and financial-scarcity items.

#### 3.1.3 Measurement

The same scales from Study 1 were used for the manipulation check of time and financial scarcity perceptions. Achievement motivation was also assessed using the same measure as in Study 1.

### 3.2 Data analysis

Data were analyzed using SPSS 26.0 for one-way ANOVA and Mplus 8.0 for path analysis.

### 3.3 Results

#### 3.3.1 Manipulation check

Results of a one-way ANOVA revealed significant differences in time scarcity perception scores among the three groups (*F*(2, 177) = 37.32, *p* < 0.001, *η_p_^2^* = 0.30; see Table 3 for means). *Post hoc* tests indicated that participants in the time scarcity condition reported significantly higher time scarcity perception than those in the financial scarcity condition (Difference = 1.29, 95% CI [0.84, 1.75], *t*(119) = 6.71, *p* < 0.001, Cohen’s *d* = 1.22) and the control condition (Difference = 1.55, 95% CI [1.10, 2.01], *t*(119) = 8.07, *p* < 0.001, Cohen’s *d* = 1.47). No significant difference was found between the financial scarcity and control conditions (Difference = 0.26, 95% CI [−0.19, 0.72], *t*(119) = 1.36, *p* = 0.36, Cohen’s *d* = 0.25).

**Table 3:** Descriptive statistics of time and financial scarcity perceptions across three conditions in study 2.

Group	Time scarcity		Financial scarcity	
	M	SD	M	SD
Time scarcity group	6.413	0.478	4.838	1.431
Financial scarcity group	5.121	1.358	6.467	0.594
Control group	4.858	1.125	4.883	1.249

Similarly, significant group differences emerged in financial scarcity perception scores (*F*(2, 177) = 51.63, *p* < 0.001, *η_p_^2^* = 0.31; see Table 3 for means). *Post hoc* tests showed that participants in the financial scarcity condition exhibited significantly higher financial scarcity perception than those in the time scarcity condition (Difference = 1.63, 95% CI [1.13, 2.13], *t*(119) = 7.77, *p* < 0.001, Cohen’s *d* = 1.42) and the control condition (Difference = 1.58, 95% CI [1.09, 2.08], *t*(119) = 7.55, *p* < 0.001, Cohen’s *d* = 1.38). No significant difference was observed between the time scarcity and control conditions (Difference = −0.05, 95% CI [−0.54, 0.45], *t*(119) = −0.22, *p* = 0.97, Cohen’s *d* = 0.04).

These results indicate the effectiveness of both the time scarcity and financial scarcity manipulations.

#### 3.3.2 Path analysis

To replicate the analytical approach of Study 1, we represented the three-group experimental design (time-scarcity, financial-scarcity, control) in a single path model in which two dummy-coded variables were created and simultaneously entered as predictors: (1) Group of time scarcity: 1 = time-scarcity condition, 0 = all other conditions. (2) Group of financial scarcity: 1 = financial-scarcity condition, 0 = all other conditions. (3) The control group, coded 0 on both dummy variables, served as the reference group. All three groups were included in the same model. The path coefficients therefore represent the effect of being in a given scarcity condition relative to the control condition, controlling for the other scarcity manipulation. The path model simultaneously estimated the effects of the two dummy-coded scarcity variables on Ms. and Mf, with their residual covariance freely estimated. The model was just-identified. The predictors accounted for 17.9% of the variance in Ms. (*f^2^* = 0.22, a medium-to-large effect) and 29.0% of the variance in Mf (*f^2^* = 0.41, a large effect). Full path coefficients are reported in Table 4.

**Table 4:** Path coefficients for the study 2 path model.

Path	B	*β*	*SE*	*z*	*p*	95% CI for B
TS → Ms	−0.784	−0.380	0.157	−4.979	<0.001	[−1.092, −0.475]
FS → Ms	−0.932	−0.419	0.170	−5.489	<0.001	[−1.265, −0.599]
TS → Mf	1.171	0.532	0.156	7.488	<0.001	[0.864, 1.477]
FS → Mf	1.149	0.484	0.169	6.812	<0.001	[0.818, 1.479]
Ms ↔ Mf (covariance)	−0.399	−0.455	0.073	−5.480	<0.001	[−0.542, −0.257]

The results (see Figure 2) showed that both scarcity conditions heightened Mf relative to the control group, aligning with Study 1. A divergence emerged, however, regarding Ms.: both scarcity groups reported lower levels, contradicting the null and positive associations found in Study 1 for time and financial scarcity, respectively.

**Figure 2:**
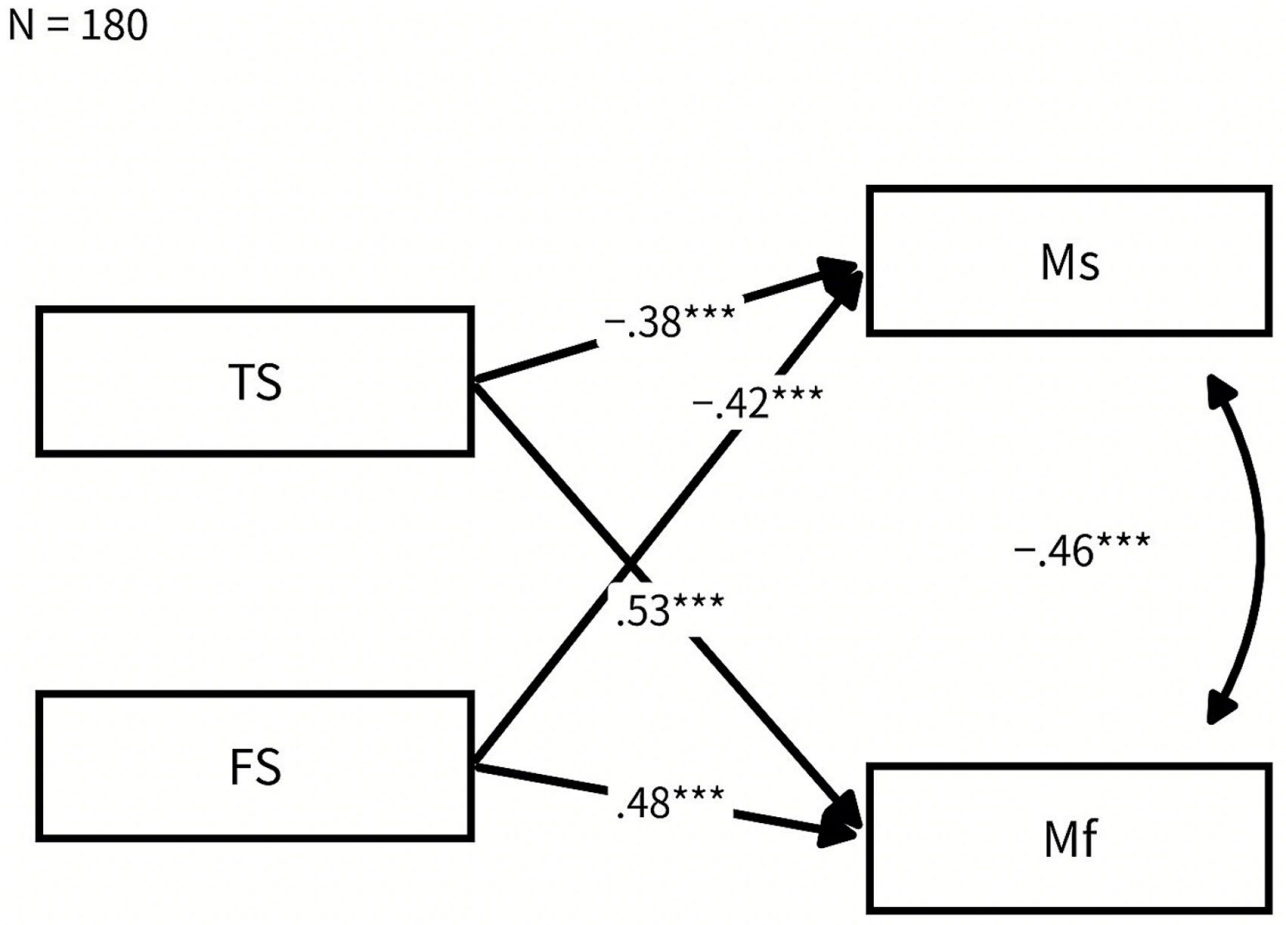
Results of path analysis in study 2 (*N* = 180). Rectangles represent measured variables; single-headed arrows represent hypothesized directional paths, with standardized path coefficients (STDYX) shown along each arrow. Solid arrows indicate statistically significant paths. The curved double-headed arrow represents the residual covariance between Ms. and Mf. TS: 1 = time-scarcity group, 0 = all other groups. FS: 1 = financial-scarcity group, 0 = all other groups. Control group coded 0 on both dummies. *p < 0.05. **p < 0.01. ***p < 0.001.

The recruited sample (*N* = 180) exceeded the sample size required by the *a priori* power analysis (*N* = 158).

### 3.4 Brief discussion

The results of Study 2 diverged from Study 1 in an important way: both time scarcity and financial scarcity significantly suppressed Ms. While we initially expected time scarcity to leave Ms. intact, its suppressive effect in this specific experimental context warrants a deeper examination of the meaning and context of the recalled experiences.

The manipulation check data showed that financial scarcity was not elevated in the time-scarcity group relative to the control group (M = 4.838 vs. M = 4.883). This effectively rules out the possibility that financial scarcity directly contaminated the time-scarcity condition. Instead, this finding suggests that recalling severe time scarcity within an unconstrained, natural context can inherently reduce Ms. A closer examination of the absolute baseline values sheds light on this contextual meaning. A control group mean of 4.883 indicates that participants in this natural recall paradigm carried a relatively high baseline level of financial constraints into the experiment. Therefore, the time-scarcity group was experiencing severe induced time scarcity (M = 6.413) within an unbuffered, demanding natural ecological context. In such a naturally stressful context, time scarcity is rarely interpreted as a positive, status-enhancing signal (Bellezza et al., 2017). Rather, it inherently signals an overwhelming workload, a loss of personal control, and burnout (Wu et al., 2025). Such perceived loss of control and ecological strain may in turn undermine the self-efficacy required for success-seeking.

This contextual dependency highlights a methodological requirement: to isolate the pure psychological signature of a specific resource scarcity from general ecological strain, it is necessary to actively anchor the non-target resource at a neutral baseline. To resolve this contextual ambiguity and precisely disentangle the effects, we designed Study 3 using an experimental control paradigm to neutralize non-target baselines.

## 4 Study 3: the effects of perceived pure time scarcity and financial scarcity on achievement motivation

Study 3 employed a one-way between-subjects design with four conditions: time-scarcity-only group, financial-scarcity-only group, dual-scarcity group (experiencing both time and financial scarcity), and control group. Participants were randomly assigned to one of these conditions. The experimental procedure consisted of three sequential parts: the experimental manipulation, a manipulation check, and the measurement of achievement motivation.

### 4.1 Method

#### 4.1.1 Participants

A sensitivity power analysis was conducted using G*Power to determine the minimum detectable effect size for a single regression coefficient in a multiple linear regression with three predictors—corresponding to the three dummy-coded experimental conditions in the path model. The analysis was anchored to the smallest significant standardized coefficient observed in Study 2 (|*β*| = 0.38), with *α* = 0.05 (two-tailed) and power = 0.80. Given a planned sample of 160 and three predictors, the analysis indicated that the minimum detectable absolute standardized coefficient was |*β*| = 0.16. The planned sample was considered adequately powered to detect individual scarcity effects of a magnitude comparable to those observed in Study 2. We recruited 160 participants through “Credamo,” with 40 individuals randomly assigned to each group. The final sample had a mean age of 30.61 years (SD = 9.59, range = 19–61) and consisted of 70 men and 90 women.

#### 4.1.2 Manipulation

We adopted the situational recall paradigm from Roux et al. (2015), modifying the past experiences to be recalled in each condition to precisely induce the targeted scarcity states:

Participants in the time-scarcity-only group were instructed to recall and describe two past experiences where they experienced a shortage of time but did not lack financial resources.

Participants in the financial-scarcity-only group recalled two past experiences involving a lack of financial resources but did not lack time.

Participants in the dual-scarcity group recalled two past experiences characterized by the co-occurrence of both time and financial scarcity.

Participants in the control group recalled two neutral past experiences involving neither time nor financial scarcity.

As part of the recall task, consistent with the procedure in Study 2, all participants responded to structured prompts asking them to elaborate on their experiences, the reasons behind the situation, and their feelings at the time. Moreover, the same manipulation check items and instructions as in Study 2 were used to assess current, state-level perceived time and financial scarcity immediately after the recall task. All participants responded to both sets of items.

#### 4.1.3 Measurement

The same measures as those used in Study 2 were adopted in this study.

### 4.2 Data analysis

The data analysis procedure was consistent with that of Study 2.

### 4.3 Results

#### 4.3.1 Manipulation check

The manipulation was again found to be effective. Participants in each scarcity condition reported significantly higher levels of the corresponding scarcity perception compared to the control group and other non-target conditions, indicating the validity of the experimental manipulation.

Results of a one-way ANOVA revealed significant differences in time scarcity perception scores among the four groups (*F*(3, 156) = 141.24, *p* < 0.001, *η_p_^2^* = 0.73; see Table 5 for means). *Post hoc* tests indicated that participants in the time-scarcity-only condition reported significantly higher time scarcity perception than those in financial-scarcity-only condition (Difference = 3.54, 95% CI [2.94, 4.13], *t*(79) = 15.44, *p* < 0.001, Cohen’s *d* = 3.45) and the control condition (Difference = 3.33, 95% CI [2.73, 3.92], *t*(79) = 14.51, *p* < 0.001, Cohen’s *d* = 3.25), participants in dual-scarcity condition reported significantly higher time scarcity perception than those in financial-scarcity-only condition (Difference = 3.33, 95% CI [2.74, 3.93], *t*(79) = 14.54, *p* < 0.001, Cohen’s *d* = 3.25) and the control condition (Difference = 3.12, 95% CI [2.52, 3.71], *t*(79) = 13.61, *p* < 0.001, Cohen’s *d* = 3.04). No significant difference was found between the time-scarcity-only and dual-scarcity conditions (Difference = 0.21, 95% CI [−0.39, 0.80], *t*(79) = 0.90, *p* = 0.81, Cohen’s *d* = 0.20), between control and financial-scarcity-only conditions (Difference = 0.21, 95% CI [−0.38, 0.81], *t*(79) = 0.93, *p* = 0.79, Cohen’s *d* = 0.21).

**Table 5:** Descriptive statistics of time and financial scarcity perceptions across four conditions in study 3.

Group	Time scarcity		Financial scarcity	
	M	SD	M	SD
Time-scarcity-only group	6.175	0.464	2.638	1.430
Financial-scarcity-only group	2.638	1.266	6.319	0.558
Dual-scarcity group	5.969	0.569	6.200	0.558
Control group	2.850	1.434	2.950	1.545

Similarly, significant group differences emerged in financial scarcity perception scores (*F*(3, 156) = 127.29, *p* < 0.001, *η_p_^2^* = 0.71; see Table 5 for means). *Post hoc* tests indicated that participants in the financial-scarcity-only condition reported significantly higher financial scarcity perception than those in time-scarcity-only condition (Difference = 3.68, 95% CI [3.03, 4.33], *t*(79) = 14.64, *p* < 0.001, Cohen’s *d* = 2.89) and the control condition (Difference = 3.37, 95% CI [2.72, 4.02], *t*(79) = 13.40, *p* < 0.001, Cohen’s *d* = 3.00), participants in dual-scarcity condition reported significantly higher financial scarcity perception than those in time-scarcity-only condition (Difference = 3.56, 95% CI [2.91, 4.22], *t*(79) = 14.17, *p* < 0.001, Cohen’s *d* = 3.17) and the control condition (Difference = 3.25, 95% CI [2.60, 3.90], *t*(79) = 12.93, *p* < 0.001, Cohen’s *d* = 2.90). No significant difference was found between the financial-scarcity-only and dual-scarcity conditions (Difference = 0.12, 95% CI [−0.53, 0.77], *t*(79) = 0.47, *p* = 0.97, Cohen’s *d* = 0.11), between control and time-scarcity-only conditions (Difference = 0.31, 95% CI [−0.34, 0.97], *t*(79) = 1.24, *p* = 0.60, Cohen’s *d* = 0.28).

#### 4.3.2 Path analysis

Similar to Study 2, the four-group experimental design (time-scarcity-only, financial-scarcity-only, dual-scarcity, and control group) was represented in a single-path model in which three dummy variables were created and simultaneously included as predictor variables in the model: (1) Group of time-scarcity-only: 1 = time-scarcity-only condition, 0 = all other conditions. (2) Group of financial-scarcity-only: 1 = financial-scarcity-only condition, 0 = all other conditions. (3) Group of dual-scarcity: 1 = dual-scarcity condition, 0 = all other conditions. (4) The control group, coded 0 on all three dummy variables, served as the reference group. All four groups were included in the same model. The path coefficients therefore represent the effect of being in a given scarcity condition relative to the control condition, controlling for the other two manipulations. The path model simultaneously estimated the effects of the three dummy-coded scarcity variables on Ms. and Mf. The model was just-identified. The four experimental conditions accounted for 6.6% of the variance in Ms. and 13.6% of the variance in Mf. Full path coefficients are reported in Table 6.

**Table 6:** Path coefficients for the study 3 path model.

Path	B	*β*	*SE*	*z*	*p*	95% CI for B
Scarcity → Ms						
TS → Ms	−0.413	−0.168	0.230	−1.794	0.073	[−0.864, 0.038]
FS → Ms	−0.471	−0.192	0.230	−2.048	0.041	[−0.922, −0.020]
TFS → Ms	−0.767	−0.312	0.230	−3.335	0.001	[−1.218, −0.316]
Scarcity → Mf						
TS → Mf	0.871	0.320	0.245	3.558	<0.001	[0.391, 1.351]
FS → Mf	0.708	0.261	0.245	2.894	0.004	[0.228, 1.188]
TFS → Mf	1.183	0.435	0.245	4.835	<0.001	[0.703, 1.663]
Residual covariance						
Ms ↔ Mf	−0.810	−0.720	0.110	−7.391	<0.001	[−1.026, −0.594]

The results (see Figure 3) yielded three findings that clarify the distinct roles of each scarcity type and address the prior inconsistencies.

**Figure 3:**
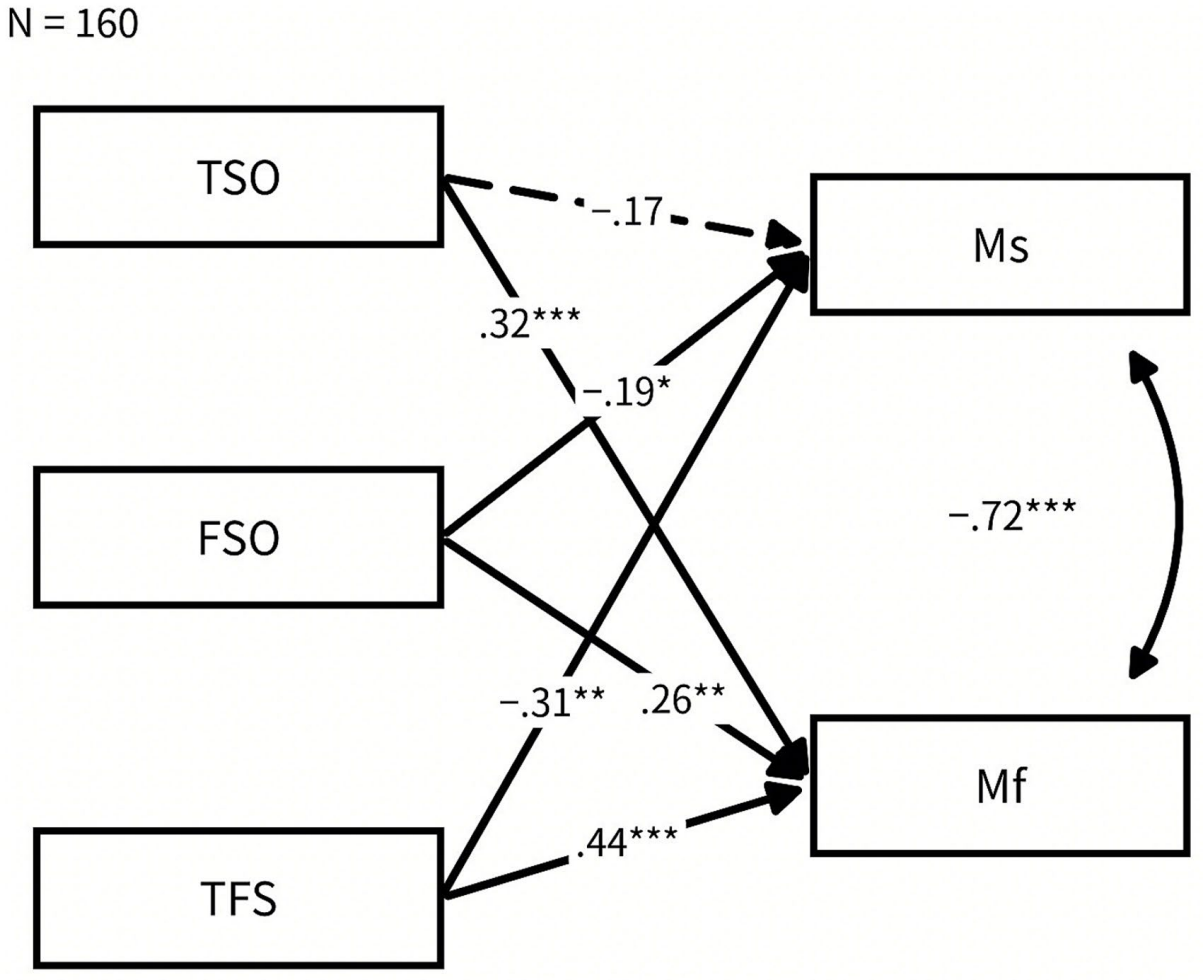
Results of path analysis in study 3 (*N* = 160). Rectangles represent measured variables; single-headed arrows represent hypothesized directional paths, with standardized path coefficients (STDYX) shown along each arrow. Solid arrows indicate statistically significant paths; the dashed arrow indicates a non-significant path. The curved double-headed arrow represents the residual covariance between Ms. and Mf. TSO: 1 = time-scarcity-only group, 0 = all other groups. FSO: 1 = financial-scarcity-only group, 0 = all other groups. TFS: 1 = dual-scarcity group, 0 = all other groups. Control group coded 0 on all dummies. *p < 0.05. **p < 0.01. ***p < 0.001.

Convergent Effect on Mf: Consistent with the results of Studies 1 and 2, participants in both the time-scarcity-only and financial-scarcity-only groups showed significantly higher Mf compared to the control group.

Divergent Effect on Ms.: A key distinction emerged in Ms. Whereas the financial-scarcity-only group showed a significant decrease, the time-scarcity-only group did not show a significant decrease, a pattern largely consistent with that initially discovered in Study 1.

The Dual-Scarcity Group: The dual-scarcity group exhibited a motivational profile similar to that of the financial-scarcity-only group, characterized by both heightened Mf and diminished Ms. This finding indicates that when the two scarcities are experienced simultaneously, the resulting motivational profile is dominated by financial scarcity, which helps to explain why the effect of time scarcity on Ms. may be obscured in naturalistic settings.

Moreover, the sensitivity power analysis indicated a minimum detectable absolute standardized coefficient of |*β*| = 0.16 for individual paths in the Study 3 model. All three scarcity conditions significantly predicted Mf (|*β*| = 0.261–0.435, all ps < 0.01). For Ms., the financial-scarcity-only (*β* = −0.192, *p* = 0.041) and dual-scarcity (*β* = −0.312, *p* = 0.001) conditions showed significant suppression, whereas the time-scarcity-only effect (*β* = −0.168, *p* = 0.073) did not reach significance. This pattern suggests that the suppressive effect of time scarcity on Ms., if present, is smaller than that of financial scarcity and may require a larger sample to be reliably detected.

#### 4.3.3 *Post-hoc* comparisons between dual-scarcity and single-scarcity conditions

To determine whether the dual-scarcity condition produced effects significantly different from those of the scarcity-only conditions, we conducted post-hoc comparisons of the unstandardized path coefficients using the Model Constraint procedure in Mplus. The results are summarized in Table 3 of Supplementary Material C. Of the four comparisons, only one reached significance: the dual-scarcity group showed a significantly greater increase in Mf compared to the financial-scarcity-only group (ΔB = 0.519, *p* = 0.037). The remaining three contrasts did not reach significance: dual-scarcity versus time-scarcity on Mf (ΔB = 0.338, *p* = 0.176), and dual-scarcity versus either single-scarcity group on Ms. (ΔB = −0.383, *p* = 0.119, dual-scarcity vs. time-scarcity; ΔB = −0.342, *p* = 0.164, dual-scarcity vs. financial-scarcity).

### 4.4 Brief discussion

These results support the “Convergent-Divergent” model. They are consistent with the idea that while generalized cognitive strain may elevate Mf, only the specific, potentially self-threatening context of financial scarcity appears to suppress Ms. We note, however, that cognitive load was not directly manipulated or measured in the present studies. The convergent elevation of Mf is therefore best interpreted as consistent with, rather than direct evidence for, a general cognitive-strain account; our orthogonal design separates the two resource-scarcity categories from one another, rather than isolating the semantic content of scarcity from cognitive load.

## 5 Discussion

The present research was designed to move beyond the “generality hypothesis” of the scarcity mindset and provide a more nuanced account of how different resource environments affect the architecture of human achievement motivation. Across three studies, using both correlational and experimental methods, we found consistent evidence for a “Convergent-Divergent” model of motivation under scarcity. Our findings indicate that the cognitive system does not respond to all forms of resource deprivation uniformly; instead, it may be sensitive to the specific social and psychological meaning of the scarce resource.

### 5.1 Theoretical implications: refining the psychology of scarcity

Our primary theoretical contribution is the refinement of scarcity theory from a social cognitive perspective. A key distinction between time and financial scarcity emerged in the present research: while both are functionally similar in triggering an adaptive defensive orientation (a convergent increase in Mf), financial scarcity appears to exert a distinctive dampening effect on advancement-oriented goals (a divergent decrease in Ms).

The convergent finding—that both time and financial scarcity increase Mf—is consistent with broader theories of threat and with empirical research on related stressors (He et al., 2024; Wu et al., 2025). When the cognitive system is under strain or perceives a potential threat to well-being (whether from a deadline, an unpaid bill, or simple mental exhaustion), it adaptively shifts into a prevention-focused, self-protective mode (Carroll et al., 2023; Civai et al., 2024; van der Veer et al., 2024). This is a functional, short-term response designed to conserve resources in a challenging environment. This offers a theoretical explanation for why individuals under pressure may tend to become more cautious and vigilant.

The divergent finding is where our model makes its most specific prediction. The suppression of Ms. was specific to the experience of financial scarcity in controlled experimental contexts. This result points to the role of social meaning and self-perception. Financial scarcity may operate as more than just a resource problem; it can act as an identity-threatening social signal (Noordewier et al., 2025). In a world where financial worth is often conflated with personal worth, a lack of financial resources can activate a cascade of negative self-evaluations (Kristoffersen et al., 2024): feelings of incompetence, low social status, and hopelessness about the future (Liang et al., 2025). This identity-threatening experience may undermine the foundations of approach motivation—such as self-efficacy and optimism—potentially suppressing the motivation to strive for success.

In contrast, the psychological impact of time scarcity depends on its ecological context. While being rushed and overwhelmed is stressful, its social meaning can be ambiguous or even positive. As observed in Study 3, when anchored to a neutral baseline, feeling time-poor may not carry the same stigma as feeling financially-poor, leaving the internal drive for achievement intact. However, as shown in Study 2, when time scarcity is experienced within an unconstrained, demanding natural context (where baseline financial pressure is relatively high), it can signal an overwhelming workload and a marked loss of personal control (Wu et al., 2025), which may in turn undermine Ms. This finding is informative, as it suggests that the core ambition and desire for growth can persist through periods of high temporal demand provided the context does not induce severe ecological strain, whereas financial precarity poses a more direct threat to that ambition.

### 5.2 Methodological contributions: from discrepancy to purification and interaction effects

The methodology of this study follows established practices in scarcity psychology. Building on Study 2, Study 3, the present study not only validated our core hypotheses but also explored the cumulative effects of resource deprivation by comparing “single scarcity” against “dual scarcity.”

Study 3 moved beyond a simple binary comparison by utilizing an orthogonal scarcity manipulation. A methodological question arises when interpreting the differences between Study 2 and Study 3: Did the explicit instruction in Study 3 (e.g., lacking time but *not lacking* finances) merely “purify” the target scarcity, or did it actively induce a state of “relative sufficiency” that functioned as a psychological buffer?

A close examination of the within-study control baselines resolves this ambiguity. In Study 2’s unconstrained recall, the control group exhibited a naturally high baseline of concurrent pressures (scarcity scores ≈ 4.8). Consequently, time scarcity in Study 2 was experienced within an unbuffered, demanding ecological context. In contrast, the Study 3 control group, instructed to recall events lacking neither resource, established a neutral, zero-scarcity baseline (≈2.9). Of note, the non-target scarcity scores in the Study 3 single-scarcity conditions (e.g., M = 2.638 for financial scarcity in the time-scarcity-only group) aligned precisely with this neutral control baseline. This indicates that the explicit manipulation successfully scrubbed the natural ecological contamination (bringing non-target scarcity down from ≈4.8 to ≈2.9) without artificially injecting a “sufficiency buffer.”

Furthermore, the asymmetrical results in Study 3 are difficult to reconcile with the buffering account. In the financial-scarcity-only group, despite the explicit neutralization of time scarcity to a non-lacking baseline (M = 2.638), the motivation to pursue success (Ms) remained significantly suppressed. This indicates that anchoring a non-target resource at a neutral baseline does not provide a protective psychological buffer. Thus, the divergent motivational effects observed in Study 3 are most consistent with the specific semantic nature of the deprived resource itself.

### 5.3 Contextual and applied perspectives

These findings have practical implications for social interventions, organizational management, and individual well-being.

(1) Social Interventions for Poverty and Financial Insecurity: Our research provides a psychological lens for understanding the pernicious effects of poverty. Policies aimed at alleviating poverty often focus on the material aspects such as providing financial assistance, food, or housing. While essential, this approach may be incomplete. Our findings suggest that financial insecurity does not just limit “buying power”; it may systematically suppress Ms. This can create a psychological trap: the very condition of poverty may diminish the aspirational drive necessary for behaviors such as challenge-taking, long-term planning, and innovation, which is essential for upward economic mobility. Interventions, therefore, must be two-pronged. Alongside material support, they should incorporate psychological components aimed at bolstering self-efficacy, fostering a growth mindset, and buffering individuals from the identity-threatening aspects of financial hardship.
(2) Workplace Dynamics and Organizational Functioning: In the modern workplace, time scarcity is an endemic contextual factor. Our results speak to a common organizational paradox: why an overstretched, “busy” workforce can simultaneously seem ambitious yet become intensely challenge-averse and resistant to innovation. The model suggests that chronic time pressure may place employees in a high-Mf state. This heightened defensive orientation could potentially make them less willing to experiment, challenge the status quo, or embrace creative but uncertain projects, even if their underlying desire for success (Ms) remains high. Leaders and managers must recognize that simply demanding longer hours or faster turnarounds can inadvertently foster a culture of prevention and caution. To maintain innovation, organizations must actively manage time pressure and create psychological safety, ensuring that the defensive mindset triggered by deadlines does not completely crowd out the approach mindset necessary for growth.

### 5.4 Limitations and future directions

Several limitations of this research point toward avenues for future inquiry.

First, our Studies 2 and 3 were conducted in controlled online experimental settings using past experiences and self-report measures. While this approach enhances internal validity and allows for precise experimental manipulation, it may limit the generalizability of our findings to real-world behavior. Relatedly, the present studies did not directly measure several of the behavioral outcomes invoked in our theoretical and applied discussions, including persistence on difficult tasks, challenge-taking in economic games, long-term planning, and observable innovative behavior. Future research should seek to replicate these findings using behavioral outcomes and in field settings to enhance external validity.

Second, and perhaps most importantly, the present studies were designed to establish the basic direct effects of scarcity on Ms. and Mf, and were not designed to directly test the mediating mechanisms through which these effects operate. Our conceptual framework proposes distinct pathways for the convergent and divergent effects: the shared threat response to scarcity (elevating Mf) may be mediated by increased anxiety or diminished sense of control, whereas the selective suppression of Ms. by financial scarcity may be mediated by reduced self-efficacy, perceived social standing, identity threat, shame, helplessness, or social comparison processes. However, neither the proposed mediating mechanisms nor cognitive load itself was directly manipulated or measured in the current investigation. Consequently, while our findings provide consistent evidence for the convergent-divergent pattern across three studies, the precise cognitive and affective mechanisms remain inferential. We encourage future research to incorporate measures of the aforementioned mediators and to employ formal mediation analyses or experimental-causal-chain designs to directly test the proposed pathways.

Third, the dual-model comparison in Study 1 yielded a theoretical insight regarding the role of subjective social standing. We deliberately avoid implying that the SSS-adjusted estimate is necessarily a “superior” test, but rather an independent one. The partial attenuation of the financial scarcity coefficient on Ms. (from *β* = −0.460 to *β* = −0.302) when SSS was introduced suggests that these constructs share theoretically meaningful variance in natural settings. This pattern suggests that while acute financial scarcity specifically suppresses Ms., it may do so partly by degrading one’s perceived social standing, indicating that SSS may function as a partial mechanistic pathway rather than a mere nuisance confounder. However, the statistically significant residual effect indicates that financial scarcity exerts an independent suppressive effect on Ms. that operates independently of individuals’ subjective social standing. This independent prediction indicates that additional mechanisms beyond social comparison, such as diminished self-efficacy or heightened identity threat, may also be involved. Furthermore, variables such as SSS, income, and education might function not only as baseline confounds or mediatorsbut also as buffers that moderate the impact of scarcity on achievement motivation. As the present research was designed to establish direct, differential effects rather than to detect interactions, we encourage future research to employ formal mediation and moderation analyses, alongside longitudinal designs, to disentangle these buffering and mediating dynamics.

Fourth, the present work focused solely on time and financial scarcity. Scarcity, however, can manifest in many other domains, such as social connection (loneliness), food security, or physical safety. Future research could explore whether the convergent-divergent model applies to these other resources. For example, does social scarcity (loneliness) primarily elevate Mf, or does it also suppress Ms. by undermining the belief that social success is possible? Expanding this framework could provide a comprehensive taxonomy of how different forms of deprivation shape the landscape of human motivation.

## 6 Conclusion

Human motivation is not a static internal trait but a dynamic system that is continuously calibrated to the opportunities and constraints of the external environment. This research indicates that the nature of this calibration is highly context-dependent. The psychological experience of scarcity is not monolithic; the mind distinguishes between the pressure of a packed calendar and the threat of an empty wallet. While any form of cognitive strain may theoretically trigger a defensive, self-protective mindset, the specific impact on advancement-oriented goals is highly context-dependent. The results suggest that financial scarcity, relative to time scarcity, more consistently impairs the motivation to pursue success across contexts, a pattern that may reflect the identity-threatening nature of financial hardship, whereas the suppressive effect of time scarcity is contingent upon the unbuffered ecological strain of the environment. By linking external social contexts to internal cognitive mechanisms, this work offers a more differentiated account of how distinct forms of resource scarcity shape achievement motivation.

## Data Availability

The raw data supporting the conclusions of this article will be made available by the authors, without undue reservation.

## References

[ref1] Adler N. E. Epel E. S. Castellazzo G. Ickovics J. R. (2000). Relationship of subjective and objective social status with psychological and physiological functioning: preliminary data in healthy white women. Health Psychol. 19, 586–592. doi: 10.1037//0278-6133.19.6.586, 11129362

[ref2] Atkinson J. W. (1957). Motivational determinants of risk-taking behavior. Psychol. Rev. 64, 359–372. doi: 10.1037/h0043445 13505972

[ref3] Bandura A. (1986). Social Foundations of Thought and action: A social Cognitive Theory. Englewood Cliffs: Prentice-Hall.

[ref4] Bandura A. (1997). Self-Efficacy: The Exercise of Control. New York: W H Freeman/Times Books/ Henry Holt & Co.

[ref5] Bandura A. (1999). “Social cognitive theory of personality,” in Handbook of Personality: Theory and Research, eds. Pervin L. A. John O. P.. 2nd ed (New York, NY: Guilford Press), 154–196.

[ref6] Bargh J. A. Chartrand T. L. (1999). The unbearable automaticity of being. Am. Psychol. 54, 462–479. doi: 10.1037/0003-066X.54.7.462

[ref7] Basileo L. D. Otto B. Lyons M. Vannini N. Toth M. D. (2024). The role of self-efficacy, motivation, and perceived support of students' basic psychological needs in academic achievement. Front. Educ. 9:1385442. doi: 10.3389/feduc.2024.1385442

[ref8] Bellezza S. Paharia N. Keinan A. (2017). Conspicuous consumption of time: when busyness and lack of leisure time become a status symbol. J. Consum. Res. 44, 118–138. doi: 10.1093/jcr/ucw076

[ref9] Boyce C. J. Brown G. D. A. Moore S. C. (2010). Money and happiness: rank of income, not income, affects life satisfaction. Psychol. Sci. 21, 471–475. doi: 10.1177/0956797610362671, 20424085

[ref10] Buttrick N. Oishi S. (2023). Money and happiness: a consideration of history and psychological mechanisms. Proc. Natl. Acad. Sci. 120:e2301893120. doi: 10.1073/pnas.2301893120, 36940338 PMC10068796

[ref11] Cannon C. Goldsmith K. Roux C. (2019). A self-regulatory model of resource scarcity. J. Consum. Psychol. 29, 104–127. doi: 10.1002/jcpy.1035

[ref12] Cannon C. Roux C. Goldsmith K. (2024). Understanding scarcity: from marketing to policy, management, and beyond. Found. Trends Mark. 19, 1–63. doi: 10.1561/1700000083

[ref13] Carroll P. J. Wichman A. L. Agler R. Arkin R. (2023). The regulation of personal security. J. Theor. Soc. Psycho. 1:7593709. doi: 10.1155/2023/7593709

[ref14] Carver C. S. Scheier M. F. (1998). On the Self-Regulation of Behavior. New York: Cambridge University Press.

[ref15] Chakraborty J. Zhang Y. Esposito A. (2025). An exploratory analysis of how perceived financial scarcity impacts learning and cognition. Curr. Psychol. 44, 15746–15757. doi: 10.1007/s12144-025-08321-w

[ref16] Civai C. Elbaek C. T. Capraro V. (2024). Why scarcity can both increase and decrease prosocial behaviour: a review and theoretical framework for the complex relationship between scarcity and prosociality. Curr. Opin. Psychol. 60:101931. doi: 10.1016/j.copsyc.2024.101931, 39442363

[ref17] de Bruijn E.-J. Antonides G. (2022). Poverty and economic decision making: a review of scarcity theory. Theor. Decis. 92, 5–37. doi: 10.1007/s11238-021-09802-7

[ref18] Dhar R. Wertenbroch K. (2000). Consumer choice between hedonic and utilitarian goods. J. Mark. Res. 37, 60–71. doi: 10.1509/jmkr.37.1.60.18718

[ref19] Elliot A. J. McGregor H. A. (2001). A 2 × 2 achievement goal framework. J. Pers. Soc. Psychol. 80, 501–519. doi: 10.1037/0022-3514.80.3.501, 11300582

[ref20] Faul F. Erdfelder E. Lang A. G. Buchner A. (2007). G*power 3: a flexible statistical power analysis program for the social, behavioral, and biomedical sciences. Behav. Res. Methods 39, 175–191. doi: 10.3758/bf03193146, 17695343

[ref21] Festinger L. (1954). A theory of social comparison processes. Hum. Relat. 7, 117–140. doi: 10.1177/001872675400700202

[ref22] Frese M. Fay D. (2001). Personal initiative: an active performance concept for work in the 21st century. Res. Organ. Behav. 23, 133–187. doi: 10.1016/S0191-3085(01)23005-6

[ref23] Gable S. L. Reis H. T. Elliot A. J. (2000). Behavioral activation and inhibition in everyday life. J. Pers. Soc. Psychol. 78, 1135–1149. doi: 10.1037//0022-3514.78.6.1135, 10870914

[ref24] He X. Qiu B. Deng Y. Wang Z. Cao X. Zheng X. . (2024). Material hardship predicts response Bias in loss-averse decisions: the roles of anxiety and cognitive control. J. Psychol. 158, 309–324. doi: 10.1080/00223980.2023.2296946, 38227200

[ref25] Hernandez-Perez J. Cruz Rambaud S. (2025). Uncovering the factors of financial well-being: the role of self-control, self-efficacy, and financial hardship. Future Bus. J. 11:70. doi: 10.1186/s43093-025-00498-7

[ref26] Higgins E. T. (1996). “Knowledge activation: accessibility, applicability, and salience,” in Social Psychology: Handbook of Basic Principles, (New York: The Guilford Press), 133–168.

[ref27] Kahneman D. (2011). Thinking, Fast and Slow. New York: Farrar, Straus and Giroux.

[ref28] Kristoffersen I. Hoang D. Li I. W. (2024). Understanding the mental health-based poverty trap: dynamics in psychological distress and financial precariousness, and the role of self-efficacy. J. Behav. Exp. Econ. 111:102219. doi: 10.1016/j.socec.2024.102219

[ref29] Kruglanski A. W. Pierro A. Mannetti L. Higgins T. E. (2013). The distinct psychologies of “looking” and “leaping”: assessment and locomotion as the springs of action. Soc. Personal. Psychol. Compass 7, 79–92. doi: 10.1111/spc3.12015

[ref30] Li J. Wang C. King R. B. (2024). Which comes first? Modeling longitudinal associations among self-efficacy, motivation, and academic achievement. System 121:103268. doi: 10.1016/j.system.2024.103268

[ref31] Liang S. Li J. Zhang T. Zhou Y. Hao Y. Lv Y. . (2025). Being alone or together: how financially constrained individuals restore their self-esteem through consumption decisions. J. Soc. Pers. Relat. 42, 2413–2435. doi: 10.1177/02654075251339269

[ref32] Liu X. Li Y. Cao X. (2024). Bidirectional reduction effects of perceived stress and general self-efficacy among college students: a cross-lagged study. Humanit. Soc. Sci. Commun. 11:271. doi: 10.1057/s41599-024-02785-0

[ref33] Mani A. Mullainathan S. Shafir E. Zhao J. (2013). Poverty impedes cognitive function. Science 341, 976–980. doi: 10.1126/science.1238041 23990553

[ref34] McClelland D. (1961). The Achieving Society. Princeton: D. Van Norstrand Company. Inc.

[ref35] Mischel W. Shoda Y. (1995). A cognitive-affective system theory of personality: Reconceptualizing situations, dispositions, dynamics, and invariance in personality structure. Psychol. Rev. 102, 246–268. doi: 10.1037/0033-295X.102.2.246, 7740090

[ref36] Monga A. Zor O. Siddiqui R. A. (2022). “The role of time in consumer psychology,” in APA Handbook of Consumer Psychology, (Washington: American Psychological Association), 413–428.

[ref37] Mullainathan S. Shafir E. (2013). Scarcity: Why Having too Little Means so Much. New York: Times Books/Henry Holt and Co.

[ref38] Muthen L. Muthen B. (2017). Mplus: Statistical Analysis with Latent Variables: User’s Guide (Version 8). Los Angeles: Authors.

[ref39] Noordewier M. K. Doolaard F. T. Lelieveld G.-J. van Dijk E. Hilbert L. P. Marr S. . (2025). A longitudinal study on the association between financial scarcity and feelings of societal exclusion. J. Behav. Exp. Econ. 114:102319. doi: 10.1016/j.socec.2024.102319

[ref40] Nygård R. Gjesme T. (1973). Assessment of achievement motives: comments and suggestions. Scand. J. Educ. Res. 17, 39–46. doi: 10.1080/0031383730170104

[ref41] Park L. E. Ward D. E. Naragon-Gainey K. (2017). It’s all about the money (for some): consequences of financially contingent self-worth. Personal. Soc. Psychol. Bull. 43, 601–622. doi: 10.1177/0146167216689080, 28903640

[ref42] Pfund G. N. Willroth E. C. Mroczek D. K. Hill P. L. (2024). Valuing versus having: the contrary roles of valuing and having money and prestige on well-being. Soc. Psychol. Personal. Sci. 15, 275–287. doi: 10.1177/19485506231166048, 38435845 PMC10904030

[ref43] Roux C. Goldsmith K. Bonezzi A. (2015). On the psychology of scarcity: when reminders of resource scarcity promote selfish (and generous) behavior. J. Consum. Res. 42, 615–631. doi: 10.1093/jcr/ucv048

[ref44] Schisterman E. F. Cole S. R. Platt R. W. (2009). Overadjustment bias and unnecessary adjustment in epidemiologic studies. Epidemiology 20, 488–495. doi: 10.1097/ede.0b013e3181a819a1, 19525685 PMC2744485

[ref45] Shah A. K. Mullainathan S. Shafir E. (2012). Some consequences of having too little. Science 338, 682–685. doi: 10.1126/science.1222426, 23118192

[ref46] Sheehy-Skeffington J. (2020). The effects of low socioeconomic status on decision-making processes. Curr. Opin. Psychol. 33, 183–188. doi: 10.1016/j.copsyc.2019.07.043, 31494518

[ref47] So J. (2018). The Psychology of Constraints: The time Scarcity and Money Scarcity effect. (Ph.D.). University of Washington, APA PsycInfo® database.

[ref48] Tang H. Lu N. (2013). Measure invariance research of the short form of achievement motive scale in boy-girl of middle school students in Shenzhen. Adv Psychol China 3, 321–326. doi: 10.12677/AP.2013.36048

[ref49] Thulien N. S. Gastaldo D. Hwang S. W. McCay E. (2018). The elusive goal of social integration: a critical examination of the socio-economic and psychosocial consequences experienced by homeless young people who obtain housing. Can. J. Public Health 109, 89–98. doi: 10.17269/s41997-018-0029-6, 29981071 PMC6964792

[ref50] Trope Y. Liberman N. (2010). Construal-level theory of psychological distance. Psychol. Rev. 117, 440–463. doi: 10.1037/a0018963, 20438233 PMC3152826

[ref51] van der Veer A. Madern T. van Lenthe F. J. (2024). Tunneling, cognitive load and time orientation and their relations with dietary behavior of people experiencing financial scarcity – an AI-assisted scoping review elaborating on scarcity theory. Int. J. Behav. Nutr. Phys. Act. 21:26. doi: 10.1186/s12966-024-01576-9, 38439067 PMC10910771

[ref52] Vohs K. D. Mead N. L. Goode M. R. (2006). The psychological consequences of money. Science 314, 1154–1156. doi: 10.1126/science.1132491, 17110581

[ref53] Wright S. (1960). The treatment of reciprocal interaction, with or without lag, in path analysis. Biometrics 16, 423–445. doi: 10.2307/2527693

[ref54] Wu Y. Feng J. Chen W. Chen Y. (2025). The effects of time and money scarcity on regulatory focus: the mediating role of sense of control. Acta Psychol. 258:105251. doi: 10.1016/j.actpsy.2025.105251, 40617112

[ref55] Zhao J. Tomm B. M. (2018). “Psychological responses to scarcity,” in Oxford Research Encyclopedia of Psychology, (Oxford: Oxford University Press), 1–21.

