# Supplementary material for: Diversity in scarcity: financial scarcity uniquely impairs success-seeking beyond a universal surge in failure-avoidance
Source: Front Psychol. 2026 Sep 28;17:1870851. doi: 10.3389/fpsyg.2026.1870851 (PMC13655092; doi:10.3389/fpsyg.2026.1870851)
Supplement: Supplementary file 1 [file Supplementary_file_1.DOCX]

**Measurement of Time/Financial Scarcity in Study 1**

English Version

**Questionnaire of Time Scarcity**

Please read the following description carefully and rate your level of agreement with each statement based on a six-month recall period. **1 indicates strongly disagreement, 7 indicates strongly agreement, and higher numbers represent greater agreement.**

| Items | 1 | 2 | 3 | 4 | 5 | 6 | 7 |
| --- | --- | --- | --- | --- | --- | --- | --- |
| 1. My time resources are scarce. |  |  |  |  |  |  |  |
| 2. I don't have enough time. |  |  |  |  |  |  |  |
| 3. I need to protect the time resources I have. |  |  |  |  |  |  |  |
| 4. I need to acquire more time. |  |  |  |  |  |  |  |

**中文版本**

**时间匮乏感问卷**

请仔细阅读下列描述，回忆您近六个月的情况，并分别评价您对每项描述的同意程度。**1代表非常不同意，7代表非常同意，数字越大代表同意程度越高。**

|  | 1 | 2 | 3 | 4 | 5 | 6 | 7 |
| --- | --- | --- | --- | --- | --- | --- | --- |
| 1. 我的时间比较紧张。 |  |  |  |  |  |  |  |
| 2. 我没有充足的时间。 |  |  |  |  |  |  |  |
| 3. 我需要节约使用我的时间。 |  |  |  |  |  |  |  |
| 4. 我需要获取更多的时间。 |  |  |  |  |  |  |  |

English Version

**Questionnaire of Financial Scarcity**

Please read the following description carefully and rate your level of agreement with each statement based on a six-month recall period. **1 indicates strongly disagreement, 7 indicates strongly agreement, and higher numbers represent greater agreement.**

| Items | 1 | 2 | 3 | 4 | 5 | 6 | 7 |
| --- | --- | --- | --- | --- | --- | --- | --- |
| 1. My financial resources are scarce. |  |  |  |  |  |  |  |
| 2. I don't have enough money. |  |  |  |  |  |  |  |
| 3. I need to protect the financial resources I have. |  |  |  |  |  |  |  |
| 4. I need to acquire more money. |  |  |  |  |  |  |  |

**中文版本**

**金钱匮乏感问卷**

请仔细阅读下列描述，回忆您近六个月的情况，并分别评价您对每项描述的同意程度。**1代表非常不同意，7代表非常同意，数字越大代表同意程度越高。**

|  | 1 | 2 | 3 | 4 | 5 | 6 | 7 |
| --- | --- | --- | --- | --- | --- | --- | --- |
| 1. 我缺钱。 |  |  |  |  |  |  |  |
| 2. 我没有充足的金钱。 |  |  |  |  |  |  |  |
| 3. 我需要节约使用我的金钱。 |  |  |  |  |  |  |  |
| 4. 我需要获取更多的金钱。 |  |  |  |  |  |  |  |

**Measurement of Time/Financial Scarcity in Study 2 and 3**

English Version

**Questionnaire of Time Scarcity**

Please read the following descriptions carefully and, based on your current feelings, rate your level of agreement with each description. **1 indicates strongly disagreement, 7 indicates strongly agreement, and higher numbers represent greater agreement.**

| Items | 1 | 2 | 3 | 4 | 5 | 6 | 7 |
| --- | --- | --- | --- | --- | --- | --- | --- |
| 5. My time resources are scarce. |  |  |  |  |  |  |  |
| 6. I don't have enough time. |  |  |  |  |  |  |  |
| 7. I need to protect the time resources I have. |  |  |  |  |  |  |  |
| 8. I need to acquire more time. |  |  |  |  |  |  |  |

**中文版本**

**时间匮乏感问卷**

请仔细阅读下列描述，并根据您此时此刻的感受，分别评价您对每项描述的同意程度。**1代表非常不同意，7代表非常同意，数字越大代表同意程度越高。**

|  | 1 | 2 | 3 | 4 | 5 | 6 | 7 |
| --- | --- | --- | --- | --- | --- | --- | --- |
| 5. 我的时间比较紧张。 |  |  |  |  |  |  |  |
| 6. 我没有充足的时间。 |  |  |  |  |  |  |  |
| 7. 我需要节约使用我的时间。 |  |  |  |  |  |  |  |
| 8. 我需要获取更多的时间。 |  |  |  |  |  |  |  |

English Version

**Questionnaire of Financial Scarcity**

Please read the following descriptions carefully and, based on your current feelings, rate your level of agreement with each description. **1 indicates strongly disagreement, 7 indicates strongly agreement, and higher numbers represent greater agreement.**

| Items | 1 | 2 | 3 | 4 | 5 | 6 | 7 |
| --- | --- | --- | --- | --- | --- | --- | --- |
| 5. My financial resources are scarce. |  |  |  |  |  |  |  |
| 6. I don't have enough money. |  |  |  |  |  |  |  |
| 7. I need to protect the financial resources I have. |  |  |  |  |  |  |  |
| 8. I need to acquire more money. |  |  |  |  |  |  |  |

**中文版本**

**金钱匮乏感问卷**

请仔细阅读下列描述，并根据您此时此刻的感受，分别评价您对每项描述的同意程度。**1代表非常不同意，7代表非常同意，数字越大代表同意程度越高。**

|  | 1 | 2 | 3 | 4 | 5 | 6 | 7 |
| --- | --- | --- | --- | --- | --- | --- | --- |
| 5. 我缺钱。 |  |  |  |  |  |  |  |
| 6. 我没有充足的金钱。 |  |  |  |  |  |  |  |
| 7. 我需要节约使用我的金钱。 |  |  |  |  |  |  |  |
| 8. 我需要获取更多的金钱。 |  |  |  |  |  |  |  |
