# Supplementary material for: Diversity in scarcity: financial scarcity uniquely impairs success-seeking beyond a universal surge in failure-avoidance
Source: Front Psychol. 2026 Sep 28;17:1870851. doi: 10.3389/fpsyg.2026.1870851 (PMC13655092; doi:10.3389/fpsyg.2026.1870851)
Supplement: Supplementary file 2 [file Supplementary_file_2.DOCX]

**Measurement of Achievement Motivation**

English Version

**Achievement Motivation Scale – Short Form**

Please read each statement carefully and rate the extent to which it describes your actual situation. **Ratings range from 1 (completely disagree) to 5 (completely agree), with higher numbers indicating greater agreement.**

| Items | 1 | 2 | 3 | 4 | 5 |
| --- | --- | --- | --- | --- | --- |
| 1. I enjoy novel and challenging tasks, even risky ones. |  |  |  |  |  |
| 2. When the outcome is uncertain, I worry about failure. |  |  |  |  |  |
| 3. I like to persist in solving problems even when I am unsure of succeeding. |  |  |  |  |  |
| 4. The thought of taking on novel and challenging tasks makes me uneasy. |  |  |  |  |  |
| 5. I am drawn to challenging tasks. |  |  |  |  |  |
| 6. I feel anxious about tasks that I am not confident I can handle. |  |  |  |  |  |
| 7. The tasks that attract me most are those whose outcomes I cannot be certain of. |  |  |  |  |  |
| 8. I worry about failure in matters that require specific opportunities to succeed. |  |  |  |  |  |
| 9. I feel very excited and happy when facing challenges whose success is uncertain. |  |  |  |  |  |
| 10. I feel quite worried when doing things that appear rather difficult. |  |  |  |  |  |
| 11. If something cannot be understood immediately, I become interested in it. |  |  |  |  |  |
| 12. When I encounter problems I cannot understand right away, I feel anxious. |  |  |  |  |  |

**中文版本**

**成就动机量表简式版**

请仔细阅读下列描述，并分别评价每项描述符合您自己的真实情况的程度。**1代表完全不符合，5代表完全符合，数字越大代表符合程度越高。**

|  | 1 | 2 | 3 | 4 | 5 |
| --- | --- | --- | --- | --- | --- |
| 1. 我喜欢新奇的、有难度的任务，甚至是有风险的。 |  |  |  |  |  |
| 2. 在结果不明的情况下，我担心失败。 |  |  |  |  |  |
| 3. 我喜欢在我没把握解决的问题上坚持不懈地努力。 |  |  |  |  |  |
| 4. 一想到要去做那些新奇的、有难度的工作，我就感到不安。 |  |  |  |  |  |
| 5. 我会被有难度的任务所吸引。 |  |  |  |  |  |
| 6. 我对那些没有把握能胜任的工作感到忧虑。 |  |  |  |  |  |
| 7. 那些我不能确定是否能成功的工作最能吸引我 |  |  |  |  |  |
| 8. 对需要有特定机会才能解决的事，我会担心失败。 |  |  |  |  |  |
| 9. 当面对不确定是否能成功的难题时，我会感到非常兴奋和快乐。 |  |  |  |  |  |
| 10. 那些看起来相当有难度的事，我做的时候很担心。 |  |  |  |  |  |
| 11. 如果有些事不能立即弄懂，我会对它产生兴趣。 |  |  |  |  |  |
| 12. 当我遇到我不能立即弄懂的问题时，我会焦虑不安。 |  |  |  |  |  |
