# Supplementary material for: Diversity in scarcity: financial scarcity uniquely impairs success-seeking beyond a universal surge in failure-avoidance
Source: Front Psychol. 2026 Sep 28;17:1870851. doi: 10.3389/fpsyg.2026.1870851 (PMC13655092; doi:10.3389/fpsyg.2026.1870851)
Supplement: Supplementary file 3 [file Supplementary_file_3.DOCX]

**Supplemental Materials C**

**Table 1.** Demographic Characteristics Across Studies

| Variable | Study 1 (*N* = 200) | Study 2 (*N* = 180) | Study 3 (*N* = 160) |
| --- | --- | --- | --- |
| Age, *M* (*SD*) | 32.68 (6.67) | 33.69 (6.89) | 30.61 (9.59) |
| Gender (female), *n* (%) | 122 (61.0%) | 110 (61.1%) | 90 (56.3%) |
| Educational level (1–7), *M* (*SD*) | 5.23 (0.68) | 5.01 (0.71) | 5.06 (0.57) |
| Family income (1–10), *M* (*SD*) | 7.62 (1.37) | 7.36 (1.81) | 6.77 (1.61) |
| SSS (1–10), *M* (*SD*) | 5.70 (1.21) | 5.49 (1.24) | 5.27 (1.22) |

Note. Education: 1 = primary school or below, 2 = Junior high school or vocational junior high school, 3 = Senior high school / technical secondary school / skilled worker school / vocational high school, 4 = Associate degree (or diploma), 5 = Bachelor's degree, 6 = Master's degree, 7 = postgraduate or above. Income: 1 = less than CNY 3,601, 2 = CNY 3,601–7,200, 3 = CNY 7,201–14,000, 4 = CNY 14,001–30,000, 5 = CNY 30,001–50,000, 6 = CNY 50,001–100,000, 7 = CNY 100,001–200,000, 8 = CNY 200,001–300,000, 9 = CNY 300,001–500,000, 10 = CNY 500,001 and above. The observed income range was 2–10 in Study 1, and 1–10 in Studies 2 and 3. SSS = subjective socioeconomic status measured via the MacArthur Scale (Adler et al., 2000). SSS was measured in all three studies but was included as a covariate only in Study 1.

**Table 2.** Comparison of Path Coefficients With and Without Covariates (Study 1)

| Path | Model 1: No Covariates | | | Model 2: With Covariates | | |
| --- | --- | --- | --- | --- | --- | --- |
|  | B [95% CI] | *β* | *p* | B [95% CI] | *β* | *p* |
| TS → Ms | −0.008 [−0.068, 0.052] | −.016 | .801 | 0.007 [−0.052, 0.066] / / | .016 | .808 |
| FS → Ms | −0.222 [−0.283, −0.160] | −.460 | < .001 | −0.146 [−0.212, −0.079] | −.302 | < .001 |
| TS → Mf | 0.084 [0.022, 0.145] | .145 | .007 | 0.066 [0.002, 0.129] / / | .113 | .043 |
| FS → Mf | 0.368 [0.306, 0.431] | .623 | < .001 | 0.350 [0.278, 0.421] | .592 | < .001 |
| R²_Ms | .216 | | | .327 | | |
| R²_Mf | .458 | | | .480 | | |

**Table 3.** Post-hoc Comparisons Between Dual-Scarcity and Only-Scarcity Conditions in Study 3

| Contrast | ΔB | *SE* | *z* | *p* |
| --- | --- | --- | --- | --- |
| TFS vs. TS → Ms | −0.383 | 0.246 | −1.561 | .119 |
| TFS vs. FS → Ms | −0.342 | 0.246 | −1.391 | .164 |
| TFS vs. TS → Mf | 0.338 | 0.249 | 1.354 | .176 |
| TFSl vs. FS → Mf | 0.519 | 0.249 | 2.080 | .037 |

Note. ΔB = difference in unstandardized path coefficients. Negative values for Ms indicate greater suppression in the dual-scarcity group; positive values for Mf indicate greater elevation. P-values are two-tailed.
